# O8.8. NEUROCOGNITION IN ULTRA-HIGHRISK INDIVIDUALS AND RELATIONSHIP TO TRANSITION TO PSYCHOSIS, DEPRESSIVE DISORDER, AND FUNCTIONING: FINDINGS FROM THE NEURAPRO TRIAL

**DOI:** 10.1093/schbul/sby015.244

**Published:** 2018-04-01

**Authors:** Kelly Allott, Patrick McGorry, Luke Bolt, Barnaby Nelson, Connie Markulev, Hok Pan Yuen, Miriam Schaefer, Nilufar Mossaheb, Monika Schlögelhofer, Stephan Smesny, Ian Hickie, Gregor Berger, Eric Chen, Lieuwe de Haan, Dorien Nieman, Merete Nordentoft, Anita Riecher-Rössler, Swapna Verma, Andrew Thompson, Alison Yung, G Paul Amminger

**Affiliations:** 1 Orygen, The National Centre of Excellence in Youth Mental Health; 2 Orygen, The National Centre of Excellence in Youth Mental Health, The University of Melbourne; 3 La Trobe University; 4 Medical University of Vienna; 5 University Hospital, Jena; 6 Brain and Mind Centre, University of Sydney; 7 Child and Adolescent Psychiatric Service of the Canton of Zurich; 8 University of Hong Kong; 9 Academic Medical Center, University of Amsterdam; 10 Mental Health Center Copenhagen, University of Copenhagen; 11 University of Basel Psychiatric Clinics; 12 Institute of Mental Health, Singapore; 13 University of Warwick; 14 Institute of Brain Behaviour and Mental Health, University of Manchester

## Abstract

**Background:**

Neurocognitive impairments are a core feature of psychosis and major depressive disorder (MDD) and are associated with poorer functioning outcomes in these illnesses. Individuals at ultra-high risk (UHR) for psychosis have been shown to experience mild but significant cognitive impairments relative to healthy controls. Evidence suggests neurocognitive deficits are useful predictors of transition to psychotic disorder, although there is inconsistency regarding the specific domains implicated. Furthermore, depression is common in the UHR population, but the relationship between neurocognitive impairment and depression in UHR has not been investigated. The aim of this study was to examine neurocognitive functioning in UHR participants at baseline and whether neurocognitive performance is associated with i) transition to psychosis; ii) depressive disorder; and iii) functioning at medium-term follow-up (median 24 months).

**Methods:**

Secondary analysis of data collected as part of a multi-centre RCT of omega-3 fatty acids and cognitive behavioural case management (NEURAPRO) for individuals at UHR for psychosis was conducted. Baseline, 6, 12 and 24-month (median) assessments relevant to the current study included the Comprehensive Assessment of the At-Risk Mental State (CAARMS), Structured Clinical Interview for DSM-IV (SCID), Brief Assessment of Cognition for Schizophrenia (BACS), Montgomery–Åsberg Depression Rating Scale (MADRS) and Social and Occupational Functioning Assessment Scale (SOFAS). The analysis was conducted on the 287 UHR participants (129 males, 158 females) who completed these measures. Hierarchical Cox proportional hazards regression was used to examine neurocognitive predictors of transition to psychosis, while hierarchical logistic and linear regressions were used to identify neurocognitive predictors of MDD and functioning, respectively.

**Results:**

Mean z-scores at baseline indicated that the UHR participants performed on average a quarter to half a standard deviation below healthy people in most domains (range mean z=-0.24 to -0.47), except for executive functioning (mean z=0.16, SD=1.21). One hundred and nineteen (41.5%) participants met criteria for MDD at baseline. Thirty-eight (13.2%) participants transitioned to psychosis over 24 months. Results showed that poorer Executive (p=.01) and Motor (p=.03) functions were predictive of transition to psychosis over 24 months, after controlling for other clinical and treatment variables. Forty-eight (16.7%) participants met criteria for MDD at 24 months. Faster Processing Speed significantly predicted MDD at 24 months (p=.01), but failed to retain significance after controlling for other factors, with baseline and past MDD history emerging as the strongest predictors of MDD at medium-term follow-up (both p<.001). After adjustment for IQ and baseline functioning, functional outcomes at 6, 12 and 24 months were predicted by baseline Processing Speed.

**Discussion:**

These findings suggest that neurocognitive abilities are important predictors of transition to psychosis and functional outcomes within the UHR population, but hold minimal value in predicting MDD after controlling for history of MDD. Further research is needed that examines trajectory of neurocognition over time in UHR and in relation to psychopathology and functioning outcomes.

